# Pre-existing CD4 T cell help boosts antibody responses but has limited impact on germinal center, antigen-specific B cell frequencies after influenza infection

**DOI:** 10.3389/fimmu.2023.1243164

**Published:** 2023-08-30

**Authors:** Danica F. Besavilla, Laura Reusch, Josue Enriquez, Karin Schön, Davide Angeletti

**Affiliations:** ^1^ Department of Microbiology and Immunology, Institute of Biomedicine, University of Gothenburg, Gothenburg, Sweden; ^2^ SciLifeLab, Institute of Biomedicine, University of Gothenburg, Gothenburg, Sweden

**Keywords:** influenza, CD4+ T cells, B cells, antibodies, germinal center

## Abstract

The influenza virus is a persistent burden on global health, with seasonal vaccines providing incomplete protection. CD4^+^ T cells help shape B cell and antibody responses; however, the selectivity of help and the effect on various antigen-specific B cell populations have not been fully elucidated. Here, we studied the specificity, selectivity, and influence of nucleoprotein (NP) CD4^+^ T cells on the magnitude and quality of hemagglutinin (HA) and NP-specific B cells and antibody responses. We identified immunodominant peptides and showed that peptide immunization was sufficient to induce CD4^+^ cells with Th1 and Tfh phenotypes. Surprisingly, while preexisting CD4^+^ T cells enhanced the influx of total germinal center (GC) B cells in the mediastinal lymph node after infection, this was not reflected by an increase in the frequency of antigen-specific cells within the GC. Furthermore, we demonstrated that NP-specific help was able to accelerate the kinetics and magnitude of the Ab response for NP but not for HA. Overall, our results showed that pre-existing CD4^+^ T cells provide strong cognate help during immunization or infection to enhance Ab production but not antigen-specific GC or memory B cells.

## 1 Introduction

The influenza A virus (IAV) is a serious public health threat, causing significant yearly morbidity and mortality and concerns due to the potential emergence of new pandemic viruses (1–3). After infection or vaccination, most antibodies (Abs) against IAV target the surface glycoprotein hemagglutinin (HA), which undergoes constant antigenic drift (4). Therefore, seasonal vaccines targeting HA have variable efficacy and need yearly readministration (3, 4). The internal nucleoprotein (NP) is semiconserved and is an immunodominant target of CD4^+^ T cell responses (5). However, despite being widely prevalent in the human population, Abs-targeting NP has poor protective potential (6). Therefore, it is important to devise strategies to boost Abs to HA while leveraging conserved proteins within the virus.

B cells are crucial in promoting long-lasting immunity to IAV. After infection, there is a rapid burst of extrafollicular plasmablasts, followed by the establishment of long-lived germinal centers (GCs), which, in turn, continuously produce new antibody-secreting cells (ASCs) and memory cells (7–10). T follicular helper cells (Tfhs) are a crucial player in supporting the B cell response (9–11): not only do they have a pivotal role in recruiting B cells to the GC but they also drive the selection of high-affinity B cells from the GC and the differentiation of early, extrafollicular ASCs (12–14). Tfhs in mice are characterized by the expression of CXCR5 and PD1, and their frequency has been shown to strongly correlate with GC size (11, 15).

In recent decades, several studies have probed the relationship between CD4 T cells and antigen-specific humoral immunity (16–26) with the consistent finding that pre-existing CD4 T cells can substantially boost antigen-specific Ab responses (16–19, 23, 26). However, data on their influence on GC development are more contradictory. While some studies claim that primed CD4 T cells enhance overall GC magnitude (16, 18, 19), others have reported just a slight acceleration in the timing of GC formation (27). Within the GC reaction, CD4 T cells seem to have the important role of recruiting rarer B cells, i.e., when CD4 help is limiting, the diversity of B cells is lower and limited to the more immunodominant specificities (24, 26, 28). However, a study carefully linking CD4 T cells of defined specificity with the timing of appearance and magnitude of antigen-specific B cells and Abs upon influenza infection is still lacking.

With IAV, the provision of help is further complicated by the small size of the virion and intermolecular help. Intermolecular help takes place when CD4^+^ T cells help B cells of a different specificity in generating an Ab (25). Previous studies investigating T cell help during IAV infection are conflicting, with early data showing intermolecular help and later studies showing that only specific help is available upon infection (16, 17, 29). In larger DNA viruses, such as vaccinia, it is well established that T cell help is specific only for the same protein (23); on the other hand, it was shown that CD4^+^ helper T cells primed to internal components of influenza could provide help to HA-specific B cells to generate HA-specific Abs (17, 29). However, more recently, it was also reported that NP-primed CD4^+^ T helper cells help NP- but not HA-specific B cells and vice versa (16). While these studies mostly focused on Ab responses, no comprehensive study has been performed regarding the generation and antigen specificity of effector B cells. As memory B cells have been shown to be more broadly reactive, to respond to future variant viral challenges, it is important to define the role of CD4^+^ T cells in enhancing the fraction of the antigen-specific memory B cell population. This is a crucial issue that can inform the best strategies for future vaccines.

Here, we combined peptide priming with IAV infection or immunization to demonstrate the specific role of CD4 T cells in helping terminal B cells differentiate into Ab-producing cells. We showed that even after IAV infection, help is limited to cognate proteins to boost the Ab response and accelerate GC formation but has little to no effect on total antigen-specific cells within the GC and memory pools.

## 2 Materials and methods

### 2.1 Ethics

The study and all animal procedures were approved under ethical permits 1666/19, 38/23, and 3307/20 granted by the Gothenburg Regional Animal ethics committee.

### 2.2 Animals

C57BL/6 mice, all female, 6-8 weeks old, were purchased from Janvier Labs, France. B6. SJL-*Ptprc^a^ Pepc^b^/*BoyJ (CD45.1) mice were maintained in-house. Mice were sacrificed at 0, 3, 7, 10, and 14 days post-infection or immunization. Blood was collected and dispensed into serum separation tubes (Microtube 1.1 mL Z-Gel, Sarstedt). Collected tissues were transferred into separate tubes containing PBS + 1% BSA for processing.

### 2.3 Immunizations

Peptides (0.5nM; NP 45: _264_LILRGSVAHKSCLPACV_280_ ; NP 47: _276_LPACVYGPAVASGYDFE_292_ ; NP 52: _306_LLQTSQVYSLIRPNENP_322_ ; HA 13: _73_NIAGWLLGNPECDPLLP_89_ ; HA 14 _79_LGNPECDPLLPVRSWSY_95_ ; HA 15 _85_DPLLPVRSWSYIVETPN_101_ ; HA 16 _91_RSWSYIVETPNSENGIC_107_ ; HA 17 _97_VETPNSENGICYPGDFI_113_ ; HA 18 _103_ENGICYPGDFIDYEELR_119_) were mixed with LPS (0.6μg/mL) and Addavax (Invivogen). Mice were immunized subcutaneously in the footp*ad hoc*k with 30μL/mouse. Depending on the experiments, three to six mice per group were infected, and their organs were pooled when necessary, for analysis. A measurement of 5 μg of each recombinant protein in PBS, mixed 1:1 with Addavax was injected i.p. in a volume of 140μl per mouse.

### 2.4 Infections

Influenza A/Puerto Rico/8/1934 H1N1 (PR8) was used to infect C57BL/6 mice at a dose of 500 TCID_50_ per mouse in Hanks’ Balanced Salt Solution (HBSS) + 0.1% fetal calf serum (BSA) intranasally in a volume of 25μL/mouse.

### 2.5 Adoptive T cell transfer

C57BL/6 mice were primed with the immunodominant peptides (NP 45, 47, and 52, and OVA p323 control) as described under “immunization”. pLNs and spleens from five mice were pooled, and a single-cell suspension was prepared. CD4 T cells were purified with an EasySep Mouse CD4^+^ T Cell Isolation Kit (Stem Cell Technologies) according to the manufacturer´s instructions. CD4 T cells (2 x 10^6^ per mouse) were transferred i.v. and recipient mice were infected after 16 hours.

### 2.6 ELISpot assay

#### 2.6.1 IL-2 and IFN-γ ELISpot assay

C57BL/6 mice were infected as mentioned above, and 10 d.p.i., the medLN or pLN (pooled) and the spleens were collected. Red blood cells were lysed using an ACK lysing buffer (Gibco). IL-2 ELISpot (BD Bioscience, catalog: 551076) and murine IFN-γ single-color enzymatic ELISpot (ImmunoSpot, catalog: MIFNg) were performed following the manufacturer’s protocol. Briefly, 96-well ELISpot well (MilliPoreSigma or CTL ImmunoSpot) membranes were activated with 15μL/well of 70% ethanol for approximately 2 minutes followed by three PBS washes. Specific cytokine capture solutions, provided in the kits, were then added to each well at 80μl/well and incubated and sealed in a humid chamber overnight at 4°C. For the IL-2 ELISpot, plates were washed once with blocking solution, RPMI + 10%FBS + 1% Pen/Strep/L-glutamine, followed by the addition of 200ul/well-blocking solution and incubation for 2 hours at room temperature (RT). No blocking was performed on the IFN-γ plates. Before the addition of cells, plates were washed once with 150μl/well PBS, then cells were added (200,000 cells/well per medLN/pLN and 500,000 cells/well per spleen) at 100μl/well in RPMI + 10%FBS + 10μg/mL Pen/Strep. Cells were then stimulated with 6-7 pooled 13-17-mer peptides with 11-12 amino acid overlap, spanning the whole protein sequence (**Tables S1**, **S2**), in 100μl/well in RPMI media (for IL-2) or in CTL media + 2mM glutamine (for IFN-γ) and incubated in an incubator at 37°C with 5% CO_2_ for 18 hours. Plates were washed two times with deionized water and then three times with PBS + 0.05% Tween, followed by the addition of the detection antibody solution (biotinylated anti-mouse IL-2 or biotinylated anti-mouse IFN-γ) at 100μl/well and incubation for 2 hours at RT. After incubation, plates were again washed three times with PBS + 0.05% Tween followed by the addition of streptavidin-HRP/AP at a 1:1000 dilution in a volume of 100μl/well and incubated for 1 hour at RT. After washing as described above, the IL-2 plates were developed with 50μl/well BD ELISpot AEC Substrate Reagent Set (catalog: 551951) and incubated in a humid chamber for 10 minutes at RT before stopping the reaction with water, while the IFN-γ plates were developed with 80μl/well Blue Developer Solution and incubated for 15 minutes at RT before stopping the reaction with water. Plates were scanned and spots were counted using the CTL ImmunoSpot plate reader. Spot-forming cells were normalized to 10^6^ cells.

#### 2.6.2 B cell ELISpot assay

Mice were sacrificed, and organs were harvested and processed. Ninety-six-well ELIspot plates (MilliPoreSigma) were coated with 100 μl/well recombinant HA protein at 1 μg/ml concentration, recombinant NP at 0.3 μg/ml and OVA at 200 μg/ml and incubated and sealed in a humid chamber, overnight at 4°C. Plates were blocked with 100 μl of PBS with 2% BSA and incubated for a minimum of 1 hour at RT. Cells were collected from homogenized individual pLNs, medLNs and spleens were homogenized in 0.1% BSA in PBS and cells were collected and counted using a Muse cell analyzer (Merck Millipore). A total of 150,000 cells in 150μl DMEM + 10% FBS + 10μg/ml gentamicin per well were added and serially diluted 3-fold, followed by overnight incubation at 37°C. Plates were washed three times with water and PBS + 0.05% Tween, followed by the addition of 50μl anti-mouse IgG H+L HRP (Aviva Systems Biology, catalog: OARA04973) diluted 1:5000 and incubated in a humid chamber for 2 hours at RT. After a washing step, as described above, plates were developed using BD ELISpot AEC Substrate solution and incubated in a humid chamber for 10 minutes at RT before stopping the reaction. Plates were imaged using a CTL ImmunoSpot plate reader, and spots were manually counted. Spot-forming cells were normalized to 10^6^ cells.

### 2.7 Flow cytometry

Mice were sacrificed, medLNs, pLNs, and spleens were harvested, and organs were processed. Red blood cells were lysed using ACK lysing buffer. For B cell characterization, 25μL of pre-mixed extracellular antibody mixture was added to each tube and incubated for 30 minutes at 4°C. The following antibodies were used for extracellular B cell staining: 1:700 anti-mouse CD3 BV510 (BD, catalog: 563024), 1:700 anti-mouse NK1.1 BV510 (BioLegend, catalog: 108737), 1:200 anti-mouse IgM FITC (BioLegend, catalog: 406506), 1:200 anti-mouse IgD Pacific Blue (BioLegend, catalog: 405712), 1:200 anti-mouse CD38 APC-Cy7 (BioLegend, catalog: 102728), 1:200 anti-mouse GL7 Pe-Cy7 (BioLegend, catalog: 144620), and 1:200 anti-mouse B220 BV786 (BioLegend, catalog: 103246). After a wash with 1mL of FACS-buffer (DPBS + 2% FBS + 0.5mM EDTA), the supernatant was discarded and 100μL of premixed HA and NP proteins at 6.6 nM were added to each tube for 1 hour at 4°C. Samples were washed and incubated for 30 minutes at 4°C with 50μL of 1:100 streptavidin-APC (Invitrogen, catalog: 17-4317-82). Live/Dead Aqua (Invitrogen, catalog: L34966) staining was performed, and samples were fixed with paraformaldehyde (1.5%). Samples were washed and resuspended in 200μl of FACS buffer and stored at 4°C until the day of analysis.

For T cell characterization, 50μL of premixed tetramer mixture (I-Ab NP52_306-322_ APC-LLQTSQVYSLIRPNENP, I-Ab HA16_91-107_ BV412-RSWSYIVETPNSENGIC, I-Ab human CLIP_87-101_ APC/BV421 - PVSKMRMATPPLMQA) was added to each tube and incubated for 3 hours at 4°C as the first staining step when indicated. After 2h 30 min, 25μL of premixed T cell-extracellular antibody mixture was added to each tube and incubated for 30 minutes at 4°C. The following antibodies were used for extracellular T-cell characterization: 1:200 anti-mouse CD3 Per-CP-Cy5.5 (BioLegend, catalog: 100218), 1:100 anti-mouse CD4 APC-Cy7 (BioLegend, catalog: 100526), 1:100 anti-mouse CD69 BV605 (BioLegend, catalog: 104529), 1:100 anti-mouse CD44 BV786 (BD, catalog: 563736), 1:100 anti-mouse CD19 BUV395 (BD, catalog: 563557), 1:100 anti-mouse NK1.1 BV510 (BioLegend, catalog: 108737), and 1:100 anti-mouse F4/80 BV510 (BioLegend, catalog: 123135).

The following antibodies were used for Tfh vs Tfr characterization: 1:100 anti-mouse B220 BUV395 (BD, catalog: 563793), 1:100 anti-mouse PD-1 BV711 (BioLegend, catalog: 135231), 1:100 anti-mouse CXCR5 BV650 (BD, catalog: 563981), 1:100 anti-mouse CD4 APC-Cy7 (BioLegend, catalog: 100526), 1:25 anti-mouse FOXp3 AlexaFluor 647 (BioLegend, catalog: 320014), and 1:100 anti-mouse CD45.1 PE (BioLegend, catalog: 553776). Permeabilization and intracellular staining were performed after the Live/Dead stain. In detail, 250μL of FOXP3 intracellular staining buffer (Invitrogen, catalog: 00-5523-00) was added for 1 hour at 37°C. The samples were then washed with a 2mL permeabilization buffer. A measurement of 25μL of 1:500 rat anti-mouse CD16/CD32 Fc block (BD, catalog: 553142) was added for 5 minutes at RT, followed by the addition of 25μL pre-mixed intracellular antibody for 45 minutes at RT. For intracellular staining, the following Abs were used: 1:100 anti-mouse FOXP3 PE (Invitrogen, catalog: 12577382), 1:50 anti-mouse Bcl6 AlexaFluor488 (BD, catalog: 561524), 1:50 anti-mouse Tbet Pe-Cy7 (Invitrogen, catalog: 25582582), and 1:50 anti-mouse RORγT Pe-CF-594 (BD, catalog: 562684). Following incubation, the samples were washed with perm buffer and resuspended in 200μL of FACS buffer per tube. The tubes were stored at 4°C until the day of analysis. Samples were analyzed using a BD LSR Fortessa X-20 instrument. Data analysis was performed using FlowJo V10 software (TreeStar), Microsoft Excel, and GraphPad Prism.

### 2.8 ELISA

Ninety-six half-well plates were coated with recombinant protein at 1 μg/ml in PBS and incubated overnight at 4°C. Wells were blocked with 100μL 2% BSA in PBS for 1 hour at room temperature. Plates were washed three times with PBS + 0.05% Tween and incubated with twofold serially diluted sera (starting from 1:100) in PBS + 0.05% Tween for 1.5 hr at RT. After three washes, anti-mouse IgG H+L HRP (Aviva Systems Biology, catalog: OARA04973) at a 1:5000 dilution was added, and plates were incubated for 1 hr at RT. After three washes, plates were developed using TMB (Thermo Fisher, catalog: 34029) for 5 minutes at RT and subsequently blocked with H_2_SO_4_. The absorbance was measured with a TECAN Sunrise absorbance microplate reader (catalog: 16039400) at 450 nm.

#### 2.8.1 Avidity ELISA

Plates were coated as mentioned above, and only sera with an OD_450_ higher than the background at a 1:100 dilution were tested. ELISA was performed as described above with the following modifications: one dilution per sample, corresponding to 0.9x B_max_, was tested in duplicate; after incubation with serum, wells were incubated for 5 min at RT with either 6 M urea or PBS. The avidity index was calculated by dividing the average absorbance of 6 M urea-incubated wells over the average absorbance of PBS-incubated wells.

### 2.9 Statistical analysis

GraphPad Prism software was used for statistical analysis. We compared the frequency of NP tetramer^+^ cells between the NP- and OVA-immunized groups per organ using the Student’s t-test. To compare the counts of cells between the groups in the ELISpot screening analysis, we used one-way ANOVA with multiple comparisons using Dunnett correction for each individual pool and control peptide. For comparison between NP and OVA prime groups or PR8-OVA and PR8-WT groups, a two-sided unpaired Student’s *t-*test was performed. **** denotes a p-value of ≤0.0001, *** denotes a p-value of ≤0.001, ** denotes a p-value of ≤0.01, and * denotes a p-value of ≤0.05. To compare the avidity index between days 7 and 14 from the NP or OVA prime groups, two-way ANOVA and *post hoc* Tukey’s HSD at an alpha of 0.05 were performed. **** denotes a p-value of ≤0.0001 and *denotes a p-value of ≤0.05.

## 3 Results

### 3.1 Identification of CD4 immunodominant peptides after IAV infection

To determine the influence of CD4^+^ T cells on B cell responses and B cell immunodominance, we first conducted a screen to identify immunodominant T cell epitopes within the NP and HA proteins of A/Puerto Rico/8/34 (PR8) in C57BL/6 mice. We chosethese proteins because they have been identified as immunodominant CD4 targets, and NP is relatively conserved between distinct viral strains (3, 5).

We infected C57BL/6 mice intranasally with PR8, and 10 days after infection, we performed IFN-γ and IL-2 ELISpot assays with cells from individual spleens and pooled mediastinal lymph nodes (medLNs) stimulated with overlapping peptide pools spanning the whole protein (**Tables S1**, **S2**). OVA peptide p323 was used as a control. While IFN-γ is a prototypical Th1 cytokine, IL-2 is related to T-cell proliferative capacity, and importantly, IL-2-producing T cells have been shown to preferentially differentiate into Tfh cells (30, 31).

**Figure 1:**
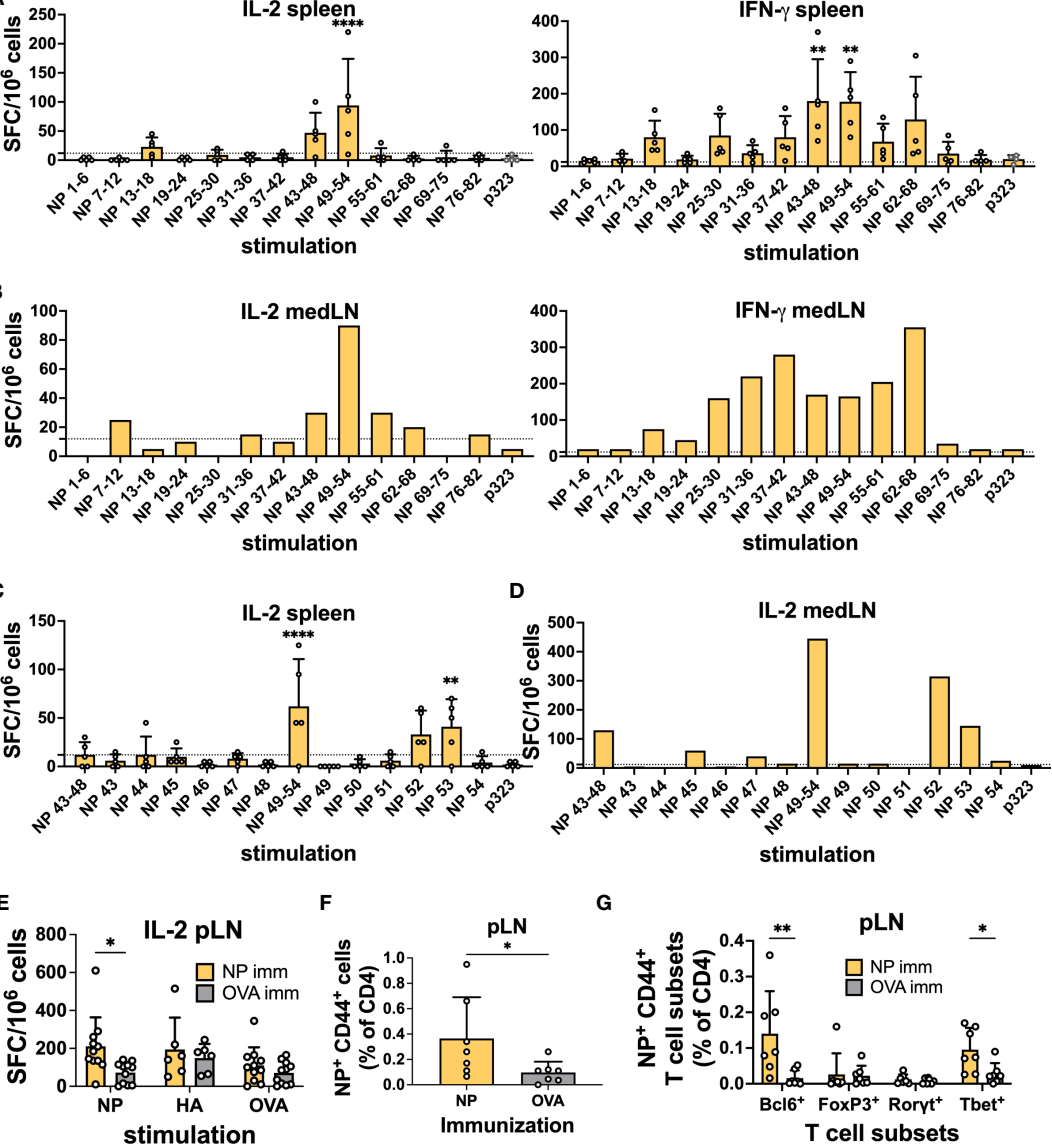
Identification of CD4 immunodominant peptides after IAV infection. **(A)** IL-2 and IFN-γ ELISpot assays with murine splenocytes and **(B)** pooled mediastinal lymph nodes (medLN) 10 days post-infection with influenza A/Puerto Rico/8/34 (PR8) mouse strain. *N* = 1, *n* = 5, where *N* corresponds to the number of independent experiments and *n* represents the number of mice per group in a given experiment. Spleens were processed individually, and medLNs were pooled. **(C)** Screening of positive NP peptide pools with the IL-2 ELISpot assay with splenocytes and **(D)** medLNs 10 days post-infection with PR8. *N* = 1, *n* = 4. Spleens were processed individually, and medLNs were pooled. The dashed line represents the average + 2*standard deviation of p323 wells as a cut-off. **(E)** Reactivity toward the positive NP peptide pool, HA peptide pool, and OVA peptide with the IL-2 ELISpot assay 10 days after immunization with NP-positive peptide pools from **Figure 1** **(A)**, with cells from the popliteal lymph node (pLN). *N* = 3, *n* = 3-5. Spot-forming cells were normalized to 10^6^ cells. **(F)** Frequency of NP52^+^ T cells (% of CD4) 10 days post-immunization with NP peptide pool or OVA peptide in the pLN. *N* = 2, *n* = 3-4. **(G)** Frequency of NP52^+^ CD44^+^ T-cell subsets (% of CD4) 10 days post-immunization with NP peptide pool or OVA peptide in the pLN. *N* = 2, *n* =3-4. Graphs represent the mean + SD. Significant differences in **Figure 1** **(A, C)** were determined with one-way ANOVA with multiple comparisons using Dunnett’s correction at an alpha of 0.05. **** denotes a p-value of ≤0.0001, *** denotes a p-value of ≤0.001, and ** denotes a p-value of ≤0.01. **(A, C)** Comparisons were performed between individual pools and the control peptide. Statistical analysis for the rest of the figure was performed with Student’s t-test. ** denotes a p-value of ≤0.01 and * denotes a p-value of ≤0.05. Data are from one experiment only **(A-D)** and are pooled from three **(E)** and two **(F, G)** independent experiments.

Upon stimulation with NP peptide pools, spot-forming cells (SFCs) were observed in the spleen samples, with NP 43-48 and 49-54 having the highest average SFCs and similar results for both cytokines (**Figure 1A**). For the medLN samples, while the IL-2 trend was very similar to that in the spleen, the IFN-γ response was much broader, with several peptides being recognized (**Figure 1B**). Subsequently, we deconvoluted the response by testing individual peptides within the positive pools and identified NP peptides 52 and 53 as sufficient to induce strong IL-2 cytokine secretion in both spleen (**Figure 1C**) and medLN (**Figure 1D**). In the medLN, peptides 45 and 47 were further identified as giving signals above the background (**Figure 1D**). For HA, we identified a single peptide pool (12–17) by ELISpot (**Figures S1A, B**); however, we were not able to deconvolute the response to a single peptide resolution as none of them gave a signal above background (data not shown). Given the similar results in the first screening, deconvolution was not carried out for IFN-γ. Overall, we identified immunodominant HA and NP PR8 CD4 T cell epitopes in BL6 mice. Our results were consistent with those previously reported from a similar screening (32).

### 3.2 The immunodominant NP peptide pool can elicit CD4 Tfh and Th1 responses.

Having identified immunodominant NP and HA peptides, we wanted to confirm their ability to induce a CD4^+^ T cell response. We immunized mice with 0.5 nM immunodominant peptide pools (NP 45, 47, and 52; HA 13-18) or OVA-p323 in the footp*ad hoc*k. At 10 days post-immunization (d.p.i.), we collected draining pLNs and spleens and tested the ability of CD4 T cells to respond to peptide stimulation by ELISpot using OVA as a stimulation control. We detected that immunization with the NP peptide pool was able to prime T cells, as measured by IL-2 and IFN-γ ELISpot (**Figure 1E** and **Figures S1C–E**). However, HA-peptide immunization failed to generate CD4 T cells above the OVA control value (**Figures S1F–I**). Similarly, when we stained T cells by flow cytometry using NP52_306-322_ and HA16_91-107_-class II tetramers, we were able to detect a clear Tetramer^+^ T cell population in the pLN (**Figure 1F**) and spleen (**Figure S1J**) only after NP immunization. In line with the ELISPOT data, HA immunization failed to yield a significant frequency of peptide-specific T cells (**Figures S1K, L**).

Furthermore, for NP-specific CD4 T cells in pLNs, we were able to classify the response obtained from immunizing with the positive peptide pools. Among NP^+^ CD44^+^ T cells, we detected a significant enrichment of Bcl6^+^ and Tbet^+^ CD4^+^ T cells compared to OVA-immunized controls (**Figure 1G**). Importantly, our results show the ability of a single NP peptide immunization to induce peptide-specific CD4 T cells that can assume Th1 and Tfh phenotypes. Given the HA-peptide prime results, we decided to focus on NP-peptide for the rest of the study.

### 3.3 After vaccination, pre-existing CD4 T cells enhance the Abs response to the same protein but not antigen-specific B cells.

The results from previous studies have been partially contradictory regarding the ability of peptide-primed CD4 T cells to help GC response and to provide intermolecular help (16, 17, 25, 29, 33). Hence, we wanted to test whether CD4 T cells induced by NP-peptide immunization could influence the humoral response to other viral proteins.

**Figure 2:**
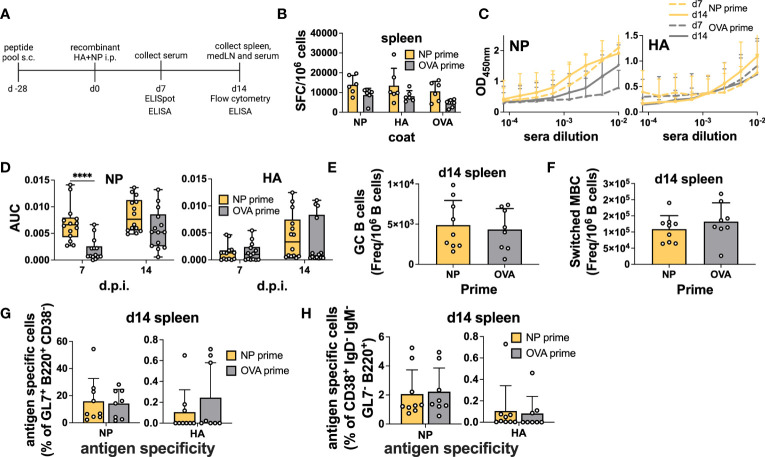
Preexisting CD4 T cells enhance cognate Abs but not antigen-specific B cells within GCs after immunization. **(A)** Experimental scheme. **(B)** B-cell ELISpot with splenocytes after positive NP peptides and OVA peptide priming followed by immunization with an equal mixture of recombinant HA and NP proteins for 7 days. Spot-forming cells were normalized to 10^6^ cells. *N* = 2, *n* = 3. **(C)** Absorbance curves of the ELISA to quantify antibody response toward recombinant NP and HA proteins after immunization at d7 and d14 and **(D)** area under the curve. *N* = 3, *n* = 3-5. **(E)** Number of GCs and **(F)** switched MBCs per 1 million B cells from the spleen. **(G)** Frequency of antigen-specific GC (GL7^+^ CD38^-^ B220^+^) and **(H)** switched memory B cells (IgD^-^ IgM^-^ GL7^-^ CD38^+^ B220^+^) 14 days post-immunization from the spleen. *N* = 3, *n* = 3-5, mice were pooled for the first experiment only. Graphs represent the mean + SD. Boxplot graph shows median +/- interquartile range **(D)**. Statistical analysis was performed with the Student’s t-test with an alpha of 0.05. **** denotes a p-value of ≤0.0001. Data are pooled from two **(B)** and three **(C-H)** independent experiments.

First, we used a simple immunization model in which NP peptide-primed (NP 45, 47, and 52) animals were subsequently vaccinated with an equal mixture of recombinant HA and NP proteins (5μg per protein) intraperitoneally (i.p.) in Addavax. Here, we did not expect any intermolecular help as the proteins are not physically attached to each other. Importantly, previous studies have shown similarities between immunodominant CD4 epitopes after infection and immunization (34). Mice were sacrificed 7 days post-immunization for B cell ELISpot and 14 days after protein immunization for flow cytometry (**Figure 2A**). Serum was collected at both time points to measure Abs. NP peptide priming slightly increased the amount of spleen antigen-specific ASCs after vaccination, as measured by ELISpot; however, no difference was detected in the response to specific proteins (**Figure 2B**). As a control, we measured ASC in pLNs, as these cells should not be affected by i.p. immunization, and confirmed that, in the majority of mice, NP-peptide immunization by itself was not sufficient to elicit detectable ASC responses (**Figure S2A**). Remarkably, in primed mice, NP-specific Abs showed accelerated kinetics, with responses already at high levels at 7 d.p.i. (**Figures 2C, D**). At 14 d.p.i., NP-Abs were still slightly higher compared to the control, but they almost plateaued from day 7 (**Figures 2C, D**). Importantly, responses to HA were not impacted (**Figures 2C, D**), as expected. Overall, when proteins are mixed, we demonstrated that CD4 T cell help is specific for the cognate protein and provides accelerated Ab kinetics.

However, when we measured total GC and antigen-specific responses by flow cytometry (**Figure S3**), we did not detect a higher frequency of total GC B cells in the spleen (**Figure 2E**). Moreover, mixed immunization was able to potently induce NP-specific GC B cells, regardless of priming, but the HA-specific response was modest (**Figure 2G**). As i.p. immunization drains to the medLN (35), we also assayed the B cell response in this organ but similarly, we were unable to detect any differences in total or antigen-specific GC B cell frequency (**Figures S2B, D**). When we analyzed memory B cells defined as CD38^+^ GL7^-^ IgD^-^ IgM^-^ B cells, we found that the presence of memory CD4 T cells did not influence the frequency of total or antigen-specific memory B cells (**Figures 2F, H** and **Figures S2C, E**).

In summary, we demonstrated that memory CD4 T cells induced by peptide immunization were able to specifically accelerate the vaccine-induced serum Ab response but did not affect GC dynamics.

### 3.4 Primed CD4^+^ T cells efficiently become Tfh cells upon infection.

**Figure 3:**
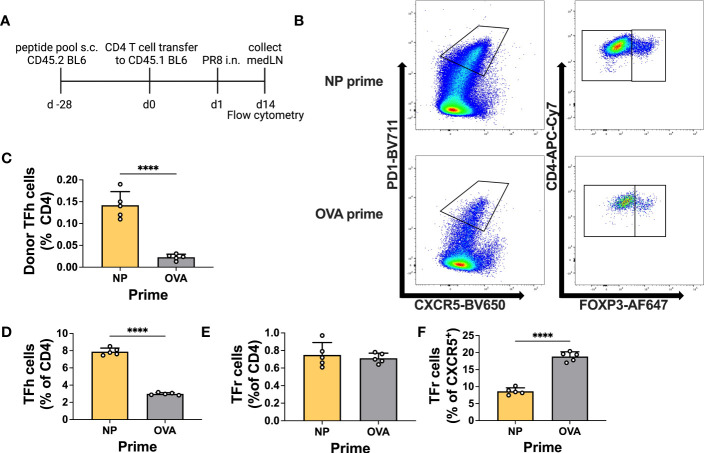
Primed CD4^+^ T cells efficiently become Tfh cells upon infection. **(A)** Experimental scheme. **(B)** Representative gating for T follicular helper (Tfh) cells or T follicular regulatory (Tfr) cells. **(C)** Frequency of donor Tfh cells (% of total CD4). **(D)** Frequency of recipient Tfh cells (PD1^+^ CXCR5^+^ CD4^+^ FOXP3^-^) and **(E)** Tfr cells (PD1^+^ CXCR5^+^ CD4^+^ FOXP3^+^). **(F)** Frequency of recipient Tfr cells (% of CXCR5^+^). Cells were obtained from medLNs. *N* = 1, *n* = 5. Graphs represent the mean + SD. Statistical analysis was performed with the Student’s t-test with an alpha of 0.05. **** denotes a p-value of ≤0.0001. Data for this figure are from one experiment only.

Prior to testing in an infection model, we decided to verify the ability of primed T cells to differentiate into Tfh cells within the medLN upon challenge. Therefore, we primed animals either with NP or control peptide and thereafter transferred total CD4 T cells to congenic mice. Recipients were challenged by i.n. infection and at 14 d.p.i. the T cells within the medLN were analyzed (**Figures 3A, B**). Overall, animals that received NP-primed CD4 T cells showed preferential expansion and differentiation of NP-specific Tfh cells compared to control-primed, transferred CD4 T cells (**Figure 3C**). In addition, animals receiving NP-primed CD4 T cells showed an overall increased frequency of Tfh cells but not Tfr cells (**Figures 3D, E**). Furthermore, we analyzed the Tfr : Tfh ratio or proportion of CXCR5^+^ cells, which are also Foxp3^+^; this measure has previously been associated with suppression of the Ab response (36). Indeed, animals that received NP-CD4 had a much lower Tfr : Tfh ratio (**Figure 3F**).

This experiment demonstrates the ability of primed T cells to differentiate into Tfh cells after infection, with the potential to influence the antibody response.

### 3.5 After infection, pre-existing CD4 T cells enhance the Ab response to the same protein and accelerate GC formation without affecting antigen-specific B cell frequency.

**Figure 4:**
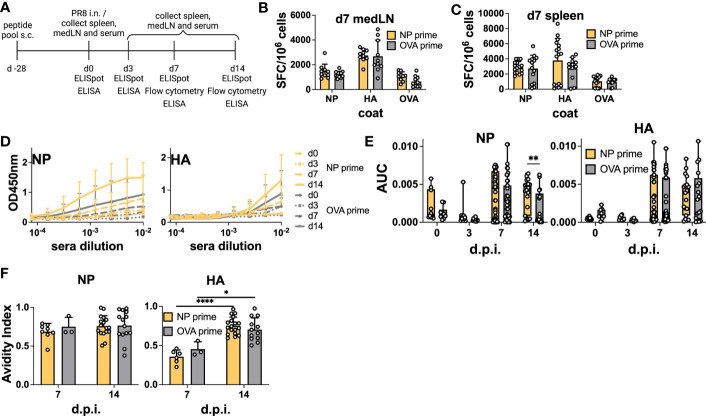
Preexisting CD4 T cells enhance cognate serum Abs but not antigen-specific ASCs after infection. **(A)** Experimental scheme. **(B)** B-cell ELISpot from positive NP peptide and OVA peptide immunization followed by PR8 infection for 7 days, with medLNs and **(C)** spleen. Spot-forming cells were normalized to 10^6^ cells. *N* = 2, *n* = 3-5. **(D)** Absorbance curves of the ELISA to quantify the antibody response toward recombinant NP and HA proteins after infection at d0, d3, d7, and d14, and **(E)** area under the curve. *N* = 1-7, *n* =5-10. **(F)** Avidity ELISA toward recombinant NP and HA proteins with sera from d7 and d14 post-infection. Only sera that had an absorbance higher than 0.2 (background) from the ELISA was used in this assay. *N*=1-4, *n* =3-5- Graphs represent the mean + SD. Boxplot graph shows median +/- interquartile range **(E)**. Statistical analysis for the rest of the figure was performed with the Student’s t-test with an alpha of 0.05. **** denotes a p-value of ≤0.0001, ** denotes a p-value ≤0.01, and * denotes a p-value of ≤0.05. Significant differences for **Figure 4** **(F)** were determined with two-way ANOVA and *post hoc* Tukey’s HSD at an alpha of 0.05. **** denotes a p-value of ≤0.0001 and *denotes a p-value of ≤0.05. Data are pooled from two **(B, C)** independent experiments. Data are representative of one (d0 and d3 D-E), seven (d7 D-E), and four experiments (d14 D-E). For the avidity ELISA, data are representative of one experiment (d7 OVA prime F), two (d7 NP prime F), and pooled four experiments (d14 NP and OVA prime F).

Having verified that NP-peptide prime was able to induce a Tfh CD4 T cell response and boost Ab responses to vaccination, we wanted to test their influence on the host humoral response after IAV infection. Here, we were particularly interested in verifying whether pre-existing NP-specific CD4 T cells would be able to help the humoral response to HA. As NP is more conserved, it would be desirable if such a response would favor neutralizing immunity to HA. Therefore, we immunized mice with the NP peptide pool and challenged them after 28 days by i.n. infection with PR8 virus (**Figure 4A**). At 0, 3, and 7 d.p.i, medLNs and spleens were harvested and processed for B cell ELISpot to monitor the emergence of antigen-specific ASCs. Day 3 ELISpot captures early extrafollicular responses, while day 7 captures a combination of extrafollicular and early GC-derived ASCs (7). Again, we analyzed both the medLN and the spleen. Previous studies have demonstrated the presence of antigen-specific B cells in both lymphoid organs after influenza infection, albeit with different dynamics (7, 8). Similar to the vaccination results, we did not detect a significant increase in NP-specific ASCs upon NP priming in any of the organs at any of the time points analyzed (**Figures 4B, C**; **Figures S4A, B**). Despite this, and in line with the data from the immunization, analysis of serum Abs demonstrated a significantly higher response in primed animals, which was specific to NP (**Figures 4D, E**). In NP-primed animals, a trend toward a higher response to NP was already observed at 7 d.p.i. and was solidified at 14 d.p.i., thus demonstrating intramolecular enhancement of the Ab response when CD4 T cell help was present (**Figures 4D, E**). HA Ab levels were not influenced by the primed CD4 T cells. As our results again showed a disconnect between ASC numbers and serum Abs, we reasoned that the total number of plasmablasts could be higher in primed animals and therefore counted total cells within the medLN. While there was a trend toward higher cellularity in the medLNs of day 7 primed animals, the number was not significantly higher (**Figure S4E**). Furthermore, we hypothesized that primed CD4 T cells could promote Abs of higher affinity. Therefore, we performed an avidity ELISA to determine the avidity index of antigen-specific Abs. We did not detect any difference in the avidity of NP-specific Abs in primed vs non-primed animals (**Figure 4F**), and while we cannot offer a definitive explanation, the results suggest that the higher Abs measured by ELISA in primed animals may be due to a slightly higher cell number possibly combined with a higher secretion rate of Abs. Interestingly, regardless of prime, the avidity of NP Abs was generally higher at 7 d.p.i, compared to HA-Abs, which showed more canonical affinity maturation (**Figure 4F**).

**Figure 5:**
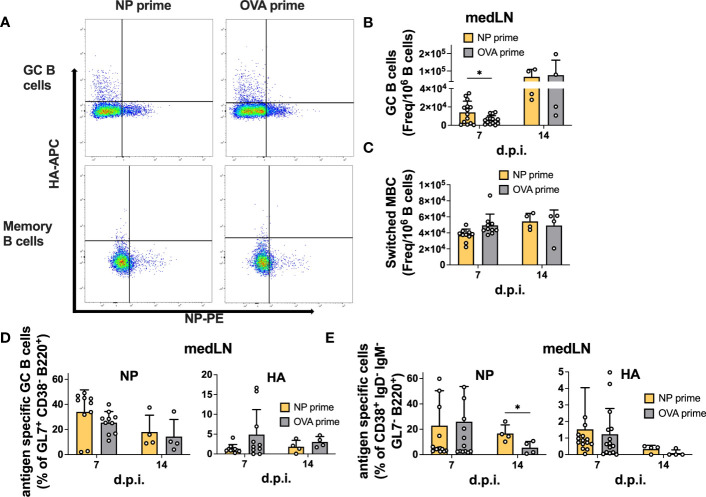
Pre-existing CD4 T cells accelerate GC formation but do not enhance antigen-specific GC or memory B cells after infection. **(A)** Representative gating for antigen-specific B-cell characterization. **(B)** Number of GCs and **(C)** switched MBCs per 1 million B cells from medLNs 7 and 14 d.p.i. **(D)** Frequency of antigen-specific GCs (GL7^+^ CD38^-^ B220^+^) and **(E)** switched memory B cells (IgD^-^ IgM^-^ GL7^-^ CD38^+^ B220^+^) in medLNs 7 and 14 d.p.i. *N* = 3-4, *n* =5-10. Statistical analysis was performed with the Student’s t-test with an alpha of 0.05. **** denotes a p-value of ≤0.0001, ** denotes a p-value ≤0.01, and * denotes a p-value of ≤0.05. For d14 flow cytometry data **(B-E)**, five mice/group were pooled per experiment (x4), while for d7 flow cytometry data **(B-E)**, all mice were individually processed per experiment (x3).

We also analyzed GC and memory B cell formation by flow cytometry (**Figure S3**). Similar to the vaccination results, we failed to detect an enhancement in total or antigen-specific GC B cells at 14 d.p.i in both the medLN (**Figures 5A, B, D**) and the spleen (**Figures S4C,F**). However, in this experiment, we also sampled 7 d.p.i. and demonstrated the role of pre-existing CD4 T cells in accelerating GC formation after IAV infection, similar to what was previously described (16). Indeed, we detected an overall increase in GC B cell frequency at 7 d.p.i. in both medLNs (**Figure 5B**) and spleens (**Figure S4C**). The frequency of switched memory B cells was unchanged (**Figure 5C** and **Figure S4D**). Importantly, when looking at antigen specificity, the overall higher GC frequency did not translate to a higher proportion of antigen-specific cells (**Figure 5D** and **Figure S4F**), an aspect that had not been investigated previously. In addition, we identified a significant increase in NP-specific memory B cells at 14 d.p.i. in the medLN (**Figure 5E**) but not in the spleen (**Figure S4G**).

Overall, our results suggest that CD4 T cell help is efficiently recalled after infection; however, while it can accelerate GC formation and size, it might not be crucial to enhance the frequency of antigen-specific B cells within the GC. Rather, it has clear effects on overall serum Ab levels and could be important for early fate decisions and terminal differentiation. Furthermore, in agreement with previous results, we show that after IAV infection, CD4 T cells do not provide intermolecular help.

## 4 Discussion

CD4^+^ T cells are crucial for antiviral immunity; however, while their role in potentiating humoral responses is well established, the fine details on how they can modulate the specificity and magnitude of B cells have not yet been fully elucidated. Indeed, previous studies yielded contradictory results in terms of the relative contribution of CD4^+^ T cells to enhancing distinct B cell subpopulations (16, 26, 27, 37). Here, we used a simple peptide-prime strategy together with IAV infection or protein vaccination to dissect the impact of preexisting CD4 T cells on humoral immunity. By combining priming and cell transfer, with detailed characterization of different populations of antigen-specific B cells, our data expand the current literature and limit the role of pre-existing CD4 T cells in boosting intramolecular Ab responses.

Given the variability of influenza HA, a viable approach to enhance humoral immunity would be to boost CD4 T cell immunity to conserved epitopes. By doing so, primed individuals would be able to better respond to novel viral variants. In this study, we set out to investigate 1) whether we could boost humoral immunity by pre-immunizing with a CD4-specific peptide pool; 2) whether all types of B cells (memory, GC, and plasmablasts) would be specifically boosted; and 3) whether the boosting would be antigen-specific. While the first goal was mainly confirmatory, given the large extent of pre-existing literature (16, 18, 23, 32), to the best of our knowledge, no data exist on which antigen-specific cells are specifically boosted by primed CD4 T cells. Indeed, several studies have noticed a correlation between CD4 T cells and heightened Ab titers (16, 27), which we confirmed here; however, whether this enhancement was dependent on increased GC entry of cognate B cells *vs* preferential expansion and differentiation to plasmablasts/plasma cells had not been elucidated. Here, we found an overall enhancement of total GC B cells early in the response (**Figure 5B**), which is in agreement with previous studies (16, 37), but not the frequency of antigen-specific B cells (**Figure 5D**). Obviously, increasing the GC size, combined with slightly increased cellularity (**Figure S4E**), has the net effect of augmenting the total number of antigen-specific B cells within the lymph node. Consistent with our work, a recent paper using transgenic CD4 T cells and HIV immunization also reported accelerated GC dynamics early after immunization but nearly unchanged GC size at later times (26). Lee et al. found the frequency of antigen-specific B cells to be increased. However, this may be because they investigated an exceedingly rare population of B cells, while in our case, the NP-specific B cell response was already robust without CD4 T cell priming (**Figure 5D**). Unfortunately, we do not have tools to dissect the epitope specificity of NP-B cells; however, we can speculate that the modulation of T cell help for GC entry is particularly relevant to broaden the range of B cell specificities recruited in the GC (28). It should be noted that other studies, either using an infection prime (19) or LAIV prime (18) and protein boost, have described a GC enhancement under these conditions: while CD4 T cells certainly contributed to other memory cells, the remaining antigen on FDC- or Ab-mediated immune complexes might all contribute to GC augmentation.

Data from serum Abs are in line with previous observations in influenza (6, 16, 18, 29) and other systems (21, 38). Importantly, these studies did not carefully analyze the dynamics of antigen-specific B cells within the GC. Surprisingly, here, we show that the increase in serum Abs is not fully linked to an increase in antigen-specific plasmablasts or GC B cells but just to a slight trend toward total higher cellularity in the medLN. While we cannot offer anything conclusive, we can suggest some possible explanations for the Ab-boost effect: 1) an increased secretion rate of Abs from each individual plasma cell, perhaps combined with the slightly higher cellularity of the medLN; 2) boosted responses in other anatomical sites (for example lungs), not analyzed here; 3) higher levels of low-affinity B cells/Abs which are not detected by ELISpot and flow cytometry but are in the ELISA; 4) accelerated GC-dependent plasmablast response in primed animals (i.e., plasmablasts could emerge between days 4 and 6, a time window we have not tested). Regardless, it appears evident that preexisting T helper cells are very efficient at boosting serum Ab responses. Future studies should address whether the heightened serum Ab levels are maintained long after infection. While this might not be relevant for clearance of the ongoing infection, it could be beneficial to prevent future infections with the same viral variant (39). Similarly, only in our infection model did we detect an increased number of memory B cells at 14 d.p.i., which was not recapitulated after immunization. We can speculate that the abundance of pre-existing CD4 T cells could also favor terminal differentiation to memory cells and GC exit.

Previous work demonstrated that Th1 cells might preferentially favor extrafollicular ASC differentiation (12), which may partially explain the results observed herein. Indeed, our peptide choice was based on IFN-γ and IL-2 ELISpot, which is consistent with standard practice. Importantly, several studies have demonstrated that these CD4 T cells can differentiate into Tfh cells (30, 31). In general, Tfh fate is generally decided within the first cell divisions, and it is mostly accepted that Tfh cells will stay within the lymph nodes, while other T helper cells will migrate to the periphery (15). IL-2 is a potent inhibitor of Tfh differentiation, but cells that produce it are poised to become Tfh cells (31). Finally, our staining of NP-primed transferred CD4 T cells confirms the ability of such cells to rapidly differentiate into Tfh cells compared to controls.

Our results also support previous work in finding no intermolecular help upon IAV infection (16, 17, 33). Works from the early nineties demonstrated that NP-specific CD4 T cells were able to support heightened production of HA-specific Abs; however, these studies were performed using nude mice and cell transfer (29). Here, by comprehensively dissecting B cell and Abs specificity, we show that no enhancement of HA-specific B cells or Abs is present despite pre-existing CD4 T cells specific for NP. Unfortunately, NP Abs are poorly protective (6) despite their wide prevalence. Therefore, taking advantage of intermolecular help would have been beneficial, given the relative conservation of NP across IAV strains (3, 5, 40). In any case, identification and exploitation of conserved immunodominant CD4 T cell epitopes on HA or NA might be a viable strategy to boost humoral immunity (18, 41). When considering HA-stem, it should be noted that this might prove difficult given the lack of helper epitopes on the stalk (24). It was our intention to study the ability of HA-specific CD4 T cells to enhance B cells specific for HA, but unfortunately, we were not able to prime the T cells. We are unsure as to why this is the case: we could speculate that HA-specific CD4 T cells are fewer in frequency and, therefore, we were not able to sufficiently stimulate them with our immunization protocol. Furthermore, we used a pool of overlapping HA peptides for immunization, and perhaps this strategy is suboptimal. Future studies should modify the peptide composition, dose, and adjuvant to try to establish a better response.

An important limitation of our study is that it was carried out in one mouse strain and with one adjuvant. It is well known that different adjuvants have the capability of polarizing T cells toward different fates (42, 43), and it may well be that with a different regimen, we could have also potentiated the GC response. Furthermore, with the current set of experiments, we are unable to exclude the possibility that NP-peptide priming promotes NP-specific memory B cell formation. These memory B cells could rapidly differentiate into ASCs and contribute to the higher Ab levels we detected here. However, given the similar number of NP-specific memory B cells with or without NP prime, we think that this is unlikely. In addition, it should be noted that some primed mice demonstrated NP-Ab titers before infection (**Figure 4E**); as LPS can activate TLR4 on B cells and stimulate plasmablast differentiation, some Ab production upon peptide priming is possible, thus enhancing the total NP response. In any case, it is important to note that our immunization strategy was similar to what others have previously adopted (16–18, 25, 26, 29, 33).

In conclusion, we present a detailed characterization of antigen-specific B cell and Ab responses in the presence of pre-existing CD4 T cells. We demonstrate that, upon IAV infection and vaccination, T cell help is specific for the cognate antigen in boosting the Ab response; however, it fails to provide an overall advantage for antigen-specific cells within the GC. Our results are of interest to researchers harnessing CD4 T cells to boost B cell immunity.

## Data availability statement

The raw data supporting the conclusions of this article will be made available by the authors, without undue reservation.

## Ethics statement

The animal study was approved by Gothenburg Regional Animal ethics committee under ethical permits 1666/19, 38/23 and 3307/20. The study was conducted in accordance with the local legislation and institutional requirements.

## Author contributions

Conceptualization: DA. Methodology: DA, DFB, LR, JE. Investigation and formal analysis: DFB, LR, JE, KS, and DA. Funding acquisition: DA. Writing—original draft: DFB and DA. Writing—reviewing and editing: LR, JE, and KS. All authors contributed to the article and approved the submitted version.
