# Supplementary material for: Pre-existing CD4 T cell help boosts antibody responses but has limited impact on germinal center, antigen-specific B cell frequencies after influenza infection
Source: Front Immunol. 2023 Aug 30;14:1243164. doi: 10.3389/fimmu.2023.1243164 (PMC10499173; doi:10.3389/fimmu.2023.1243164)
Supplement: Supplementary file 1 [file DataSheet_1.pdf]

Supplementary Material

Pre-existing CD4 T Cell help boosts antibody responses but has limited impact on germinal center, antigen-specific B cell frequencies after influenza infection.

Danica F. Besavilla[1], Laura Reusch[1]†, Josue Enriquez[1]†, Karin Schön[1], Davide Angeletti[1,2,*]

[1]Department of Microbiology and Immunology, Institute of Biomedicine, University of Gothenburg, Gothenburg, Sweden. [2]SciLifeLab, Institute of Biomedicine, University of Gothenburg, Gothenburg, Sweden

† These authors contributed equally to this work

*** Correspondence:**
Davide Angeletti
davide.angeletti@gu.se

1.1 Supplementary Figures

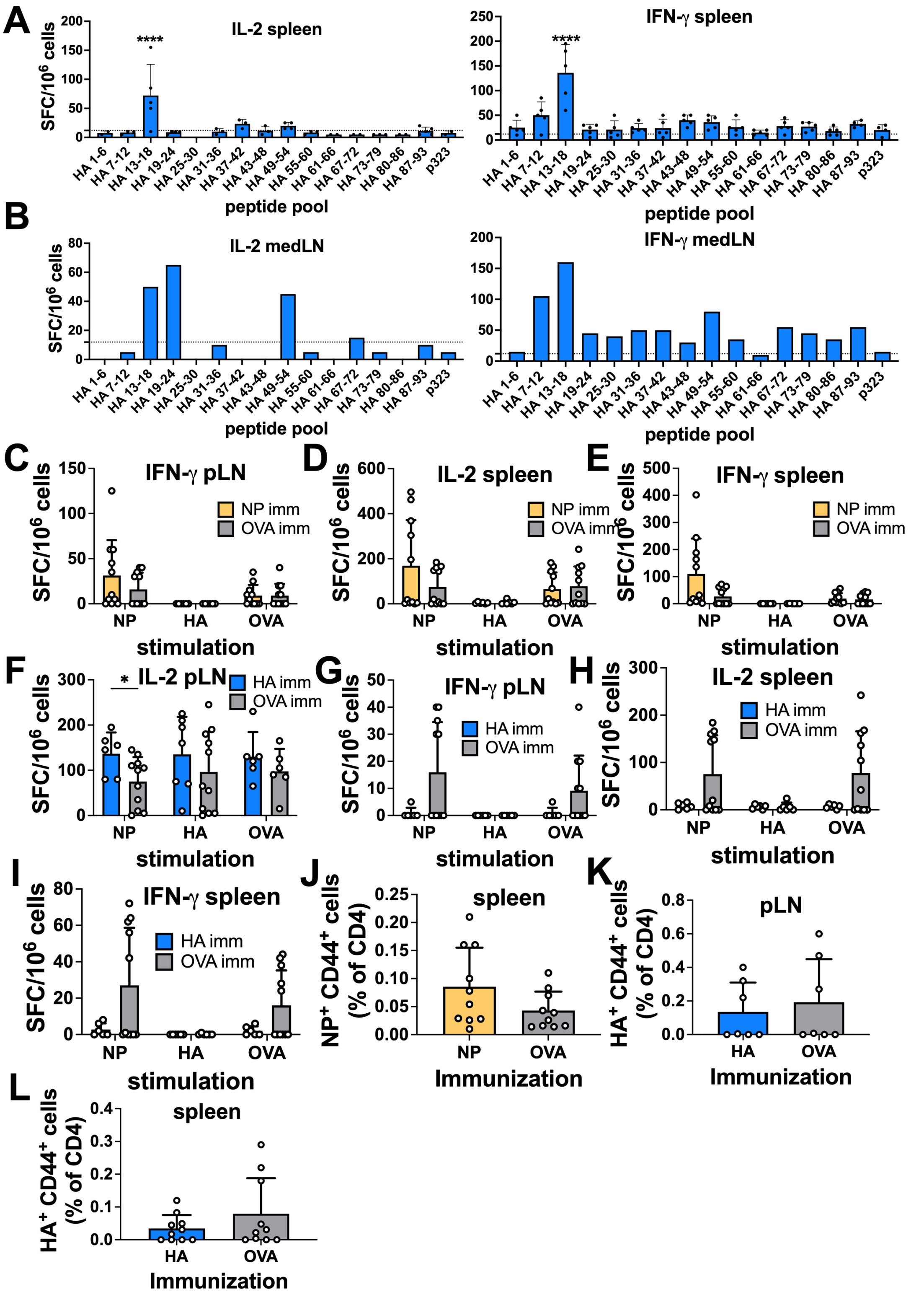

Supplementary Figure 1. Identification of CD4 immunodominant peptides after IAV infection. **(A)** IL-2 and IFN-γ ELISpot assays with murine splenocytes and **(B)** mediastinal lymph node (medLN) 10

days post infection with influenza A/Puerto Rico/8/34 (PR8) mouse strain. Dashed line represents 2*standard deviation + average of p323 wells as a cut off. $N = 1$, $n = 5$, where N corresponds to number of independent experiments and n represents the number of mice per group in a given experiment. **(C)** Reactivity towards positive NP peptide pool, HA peptide pool and OVA peptide with IL-2 and IFN-γ ELISpot assay 10 days after immunization with NP positive peptide pools, with pLN, **(D-E)** splenocytes, or HA positive peptide pools, with **(F-G)** pLN and **(H-I)** splenocytes. Spot forming cells were normalized to per 10^6 cells. $N = 3$, $n = 3$-5. **(J)** Frequency of $NP52^+$ T cell (% of CD4) 10 days post immunization with the NP positive peptides or OVA peptide in the spleen. $N = 3$, $n = 3$-4. **(K)** Frequency of $HA16^+$ T cell (% of CD4) 10 days post immunization with HA positive peptides or OVA peptide, in the pLN and **(L)** spleen. $N = 2$-3, $n = 3$-4. Graphs represent mean + SD. Significant differences for Supplementary Figures 1 **(A)** and **(B)** were determined with One-way ANOVA and post-hoc TukeyHSD at an alpha of 0.05. **** denotes a p-value of $\leq$0.0001. Statistical analysis for the rest of the figure was performed with a Student's t test. * denotes a p-value of $\leq$0.05. Data is from one experiment only **(A-B)**, is pooled of 3 **(C-I)**, 3 **(J and L)** independent experiments, and 2 **(K)** independent experiments.

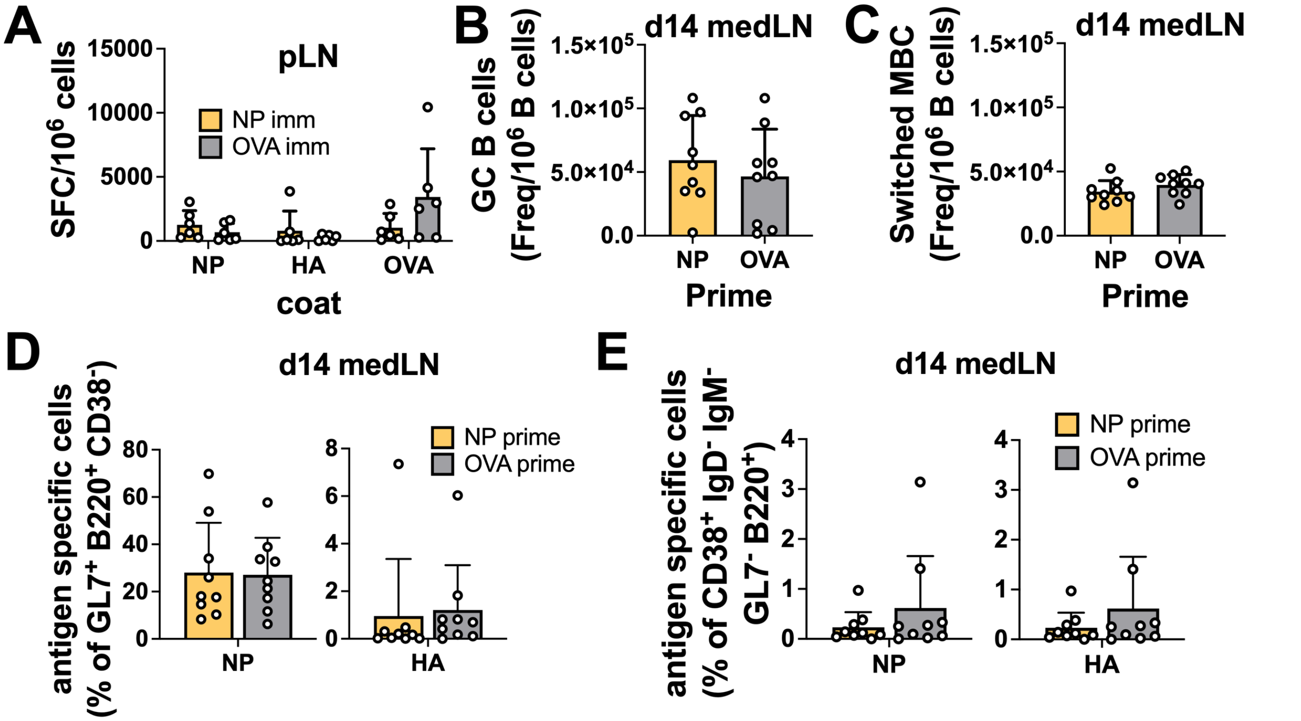

Supplementary Figure 2. Pre-existing CD4 T cells enhance cognate Abs but not antigen specific B cells, within GC, after immunization. **(A)** B cell ELISpot with pLN after NP and OVA peptide prime followed by immunization with equal mixture of recombinant HA and NP proteins for 7 days. Spot forming cells were normalized to per 10^6 cells. $N = 2$, $n = 3$. **(B)** Number of GC and **(C)** switched-MBC per 1 million B cells from the medLN 14 days post immunization. **(D)** Frequency of antigen-specific GC ($GL7^+$ $CD38^-$ $B220^+$) and **(E)** switched Memory B cells (IgD^- IgM^- $GL7^-$ $CD38^+$ $B220^+$) 14 days post immunization from medLN. $N = 3$, $n = 3$-5. Graphs represent mean + SD. Statistical analysis was performed with a Student's t test an alpha of 0.05. Data is pooled of 2 **(A)** and 3 **(B-E)** independent experiments.

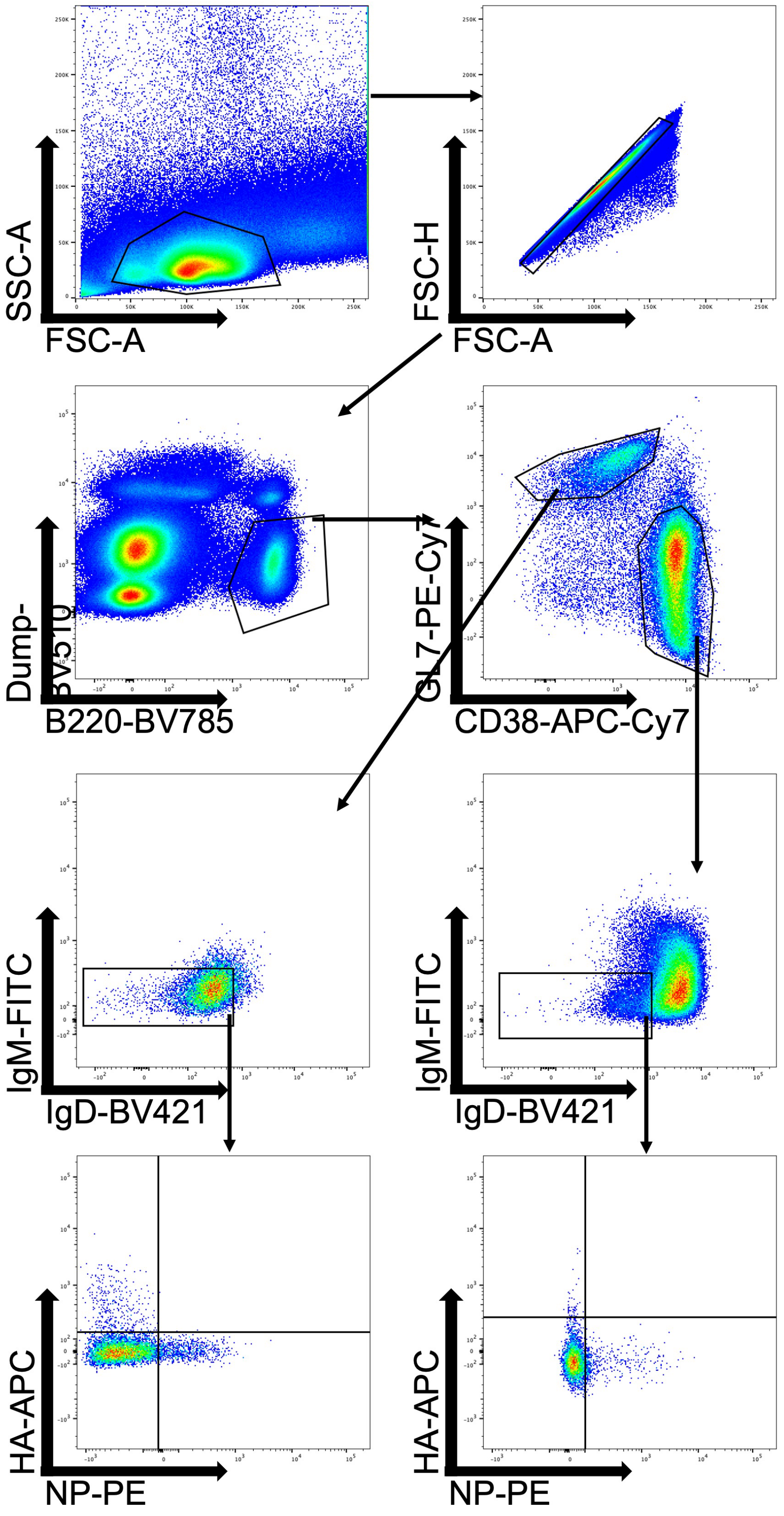

Supplementary Figure 3. Flow cytometry gating for B cell characterization and analysis.

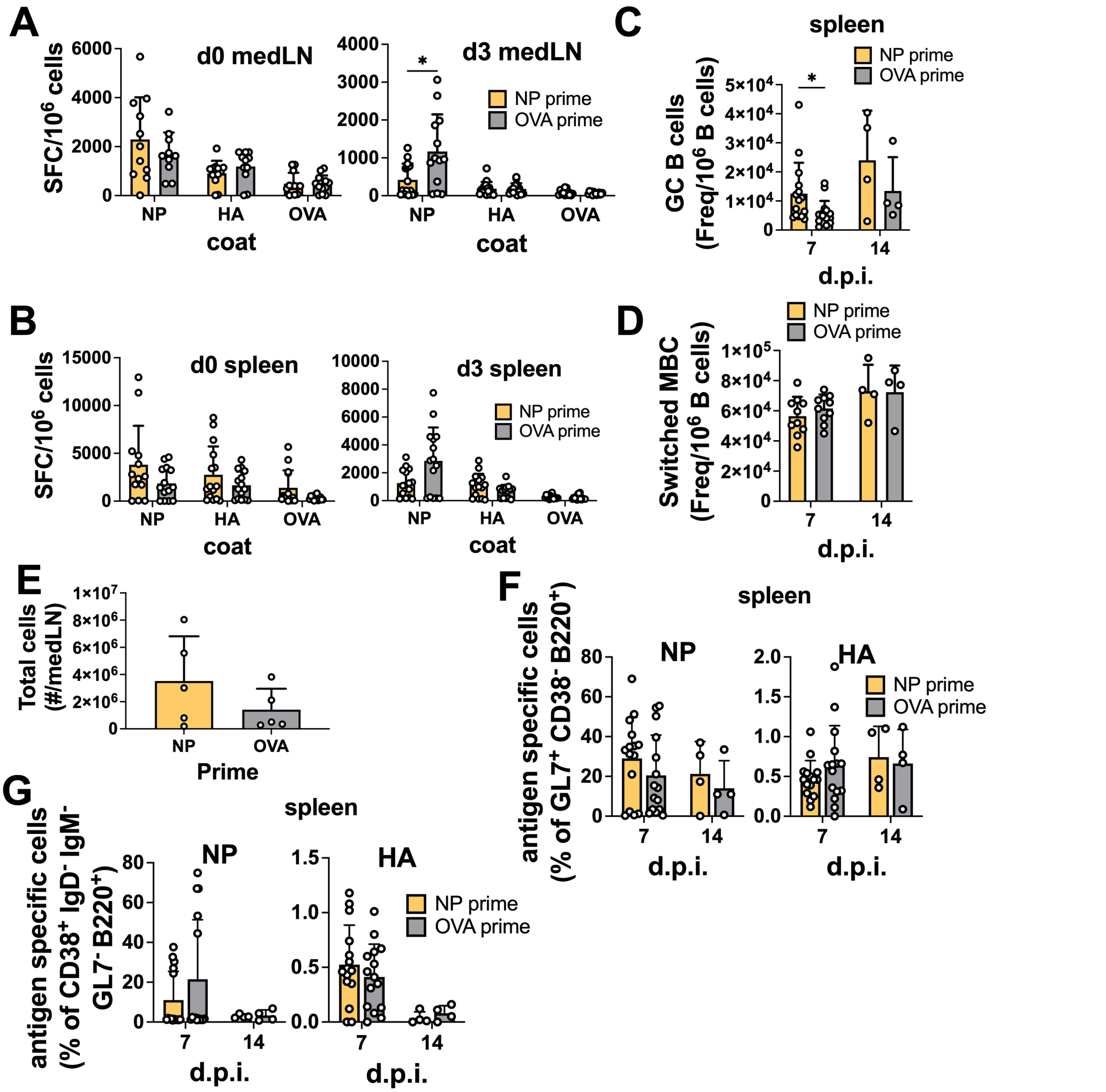

Supplementary Figure 4. Pre-existing CD4 T cells enhance cognate Abs and accelerate GC, but not antigen specific B cells, after infection. **(A)** B cell ELISpot from NP and OVA peptide immunization followed by PR8 infection at d0 and d3 medLN, **(B)** d0 and d3 spleen. Spot forming cells were normalized to per 10^6 cells. $N = 2$, $n = 5$-10. **(C)** Number of GC and **(D)** switched-MBC per 1 million B cells from the spleen 7- and 14 d.p.i.. **(E)** Total number of cells per medLN. **(F)** Frequency of antigen-specific GC ($GL7^+$ $CD38^-$ $B220^+$) and **(G)** switched-Memory B cells (IgD^- IgM^- $GL7^-$ $CD38^+$ $B220^+$) from spleen 7- and 14 d.p.i. $N = 3$-4, $n = 5$-10. Graphs represent mean + SD. Statistical analysis was performed with a Student's t test an alpha of 0.05. * denotes a p-value of ≤0.05. Data is pooled of 2 **(A-B)** independent experiments. Data is representative of 1 independent experiment **(E)**. For d14 flow cytometry data **(C, D, F, G)**, 5 mice/group were pooled per experiment (x4), while for d7 flow cytometry data **(C, D, F, G)**, all mice were individually processed per experiment (x3).

1.2 Supplementary Tables

Table S1. Table of Nucleoprotein peptides used in the study.

Peptide	Sequences
1	1 MASQGTKRYEQMETDG 17
2	7 KRSYEQMETDGERQNAT 23
3	13 METDGERQNATEIRASV 29
4	19 RQNATEIRASVGRMIGG 25
5	25 IRASVGRMIGGIGRFYI 41
6	31 RMIGGIGRFYIQMCTEL 47
7	37 GRFYIQMCTELKLNDYE 53
8	43 MCTELKLNDYEGRLIQN 59
9	49 LNDYEGRLIQNSLTIER 65
10	55 RLIQNSLTIERMVLSAF 71
11	61 LTIERMVLSAFDERRNK 77
12	67 VLSAFDERRNKYLEEHP 83
13	73 ERRNKYLEEHPSAGKDP 89
14	79 LEEHPSAGKDPKKTGGP 95
15	85 AGKDPKKTGGPIYKRVD 101
16	91 KTGGPIYKRVDGKWVRE 107
17	97 YKRVDGKWVRELVLYDK 113
18	103 KWVRELVLYDKEEIRRI 119
19	109 VLYDKEEIRRIWRQANN 125
20	115 EIRRIWRQANNGDDATA 131
21	121 RQANNGDDATAGLTHIM 137
22	127 DDATAGLTHIMIWHSNL 143
23	133 LTHIMIWHSNLNDTTYQ 149
24	139 WHSNLNDTTYQRTRALV 155
25	145 DTTYQRTRALVRTGMDP 161
26	151 TRALVRTGMDPRMCSLM 167
27	157 TGMDPRMCSLMQGSTLP 173
28	163 MCSLMQGSTLPRRSGAA 179
29	169 GSTLPRRSGAAGAAVKG 185
30	175 RSGAAGAAVKGVGTMVL 191
31	181 AAVKGVGTMVLELIRMI 197
32	187 GTMVLELIRMIKRGIND 203
33	193 LIRMIKRGINDRNFWRG 209
34	199 RGINDRNFWRGENGRKT 215
35	205 NFWRGENGRKTRIAYER 221
36	211 NGRKTRIAYERMCNILK 227
37	217 IAYERMCNILKGKFQTA 233

38	223 CNILKGKFQTAAQKAMM 239
39	229 KFQTAAQKAMMDQVRES 245
40	234 AQKAMMDQVRESRNPGN 250
41	240 DQVRESRNPGNAEIEDL 256
42	246 RNPGNAEIEDLTFLARS 262
43	252 EIEDLTFLARSALILRG 268
44	258 FLARSALILRGSVAHKS 274
45	264 LILRGSVAHKSCLPACV 280
46	270 VAHKSCLPACVYGPAVA 286
47	276 LPACVYGPAVASGYDFE 292
48	282 GPAVASGYDFEKEGYSL 298
49	288 GYDFEKEGYSLVGVDPF 304
50	294 EGYSLVGVDPFKLLQTS 310
51	300 GVDPFKLLQTSQVYSLI 316
52	306 LLQTSQVYSLIRPNENP 322
53	312 VYSLIRPNENPAHKSQL 328
54	318 PNENPAHKSQLVWMACN 334
55	324 HKSQLVWMACNSAAFED 340
56	330 WMACNSAAFEDLRVSSF 346
57	336 AAFEDLRVSSFIRGTRV 352
58	342 RVSSFIRGTRVLPRGKL 358
59	348 RGTRVLPRGKLSTRGVQ 364
60	354 PRGKLSTRGVQIASNEN 370
61	360 TRGVQIASNENMDAIVS 376
62	366 ASNENMDAIVSSTLELR 382
63	372 DAIVSSTLELRSRYWAI 388
64	378 TLELRSRYWAIRTRSGG 394
65	384 RYWAIRTRSGGNTNQQR 400
66	390 TRSGGNTNQQRASAGQI 406
67	396 TNQQRASAGQISTQPTF 412
68	402 SAGQISTQPTFSVQRNL 418
69	408 TQPTFSVQRNLPFDKTT 424
70	414 VQRNLPFDKTTIMAAFT 430
71	420 FDKTTIMAAFTGNTEGR 436
72	426 MAAFTGNTEGRTSDMRA 442
73	432 NTEGRTSDMRAEIIKMM 448
74	438 SDMRAEIIKMMESARPE 454
75	444 IIKMMESARPEEVSFQG 460
76	450 SARPEEVSFQGRGVFEL 466
77	456 VSFQGRGVFELSDERAT 472
78	462 GVFELSDERATNPIVPS 478
79	468 DERATNPIVPSFDMSNE 484

80	474 PIVPSFDMSNEGSYFFG 490
81	480 DMSNEGSYFFGDNAEEY 496
82	486 SYFFGDNAEEYDN 498

17-mer peptides with 11-12 amino acid overlap spanning the Nucleoprotein sequence.

Table S2. Table of Hemmaglutinin peptides used in the study.

Peptide	Sequence
1	1 MKANLLVLLCALAAADA 17
2	7 VLLCALAAADADTICIG 23
3	13 AAADADTICIGYHANNS 29
4	19 TICIGYHANNSTDTVDT 35
5	25 HANNSTDTVDTVLEKNV 41
6	31 DTVDTVLEKNVTVTHSV 47
7	37 LEKNVTVTHSVNLLEDS 53
8	43 VTHSVNLLEDSHNGKLC 59
9	49 LLEDSHNGKLCRLKGIA 65
10	55 NGKLCRLKGIAPLQLGK 71
11	61 LKGIAPLQLGKCNIAGW 77
12	67 LQLGKCNIAGWLLGNPE 83
13	73 NIAGWLLGNPECDPLLP 89
14	79 LGNPECDPLLPVRSWSY 95
15	85 DPLLPVRSWSYIVETPN 101
16	91 RSWSYIVETPNSENGIC 107
17	97 VETPNSENGICYPGDFI 113
18	103 ENGICYPGDFIDYEELR 119
19	109 PGDFIDYEELREQLSSV 125
20	115 YEELREQLSSVSSFERF 131
21	121 QLSSVSSFERFEIFPKE 137
22	127 SFERFEIFPKESSWPNH 143
23	133 IFPKESSWPNHNTNGVT 149
24	139 SWPNHNTNGVTAACSHE 155
25	145 TNGVTAACSHEGKSSFY 161
26	151 ACSHEGKSSFYRNLLWL 167
27	157 KSSFYRNLLWLTEKEGS 173
28	163 NLLWLTEKEGSYPKLKN 179
29	169 EKEGSYPKLKNSYVNKK 185
30	175 PKLKNSYVNKKGKEVLV 191
31	181 YVNKKGKEVLVLWGIHH 197
32	187 KEVLVLWGIHHPPNSKE 203
33	193 WGIHHPPNSKEQQNLYQ 209
34	199 PNSKEQQNLYQNENAYV 215
35	205 QNLYQNENAYVSVVTSN 221

36	211 ENAYVSVVTSNYNRRFT 227
37	217 VVTSNYNRRFTPEIAER 233
38	223 NRRFTPEIAERPKVRDQ 239
39	229 EIAERPKVRDQAGRMNY 245
40	235 KVRDQAGRMNYYWTLLK 251
41	241 GRMNYYWTLLKPGDTII 257
42	247 WTLLKPGDTIIFEANGN 263
43	253 GDTIIFEANGNLIAPMY 269
44	259 EANGNLIAPMYAFALSR 275
45	265 IAPMYAFALSRGFGSGI 281
46	271 FALSRGFGSGIITSNAS 287
47	277 FGSGIITSNASMHECNT 293
48	283 TSNASMHECNTKCQTPL 299
49	289 HECNTKCQTPLGAINSS 305
50	295 COTPLGAINSSLPYONI 311
51	301 AINSSLPYQNIHPVTIG 317
52	307 PYQNIHPVTIGECPKYV 323
53	313 PVTIGECPKYVRSAKLR 329
54	319 CPKYVRSAKLRMVTGLR 335
55	325 SAKLRMVTGLRNIPSIQ 341
56	331 VTGLRNIPSIQSRGLFG 347
57	337 IPSIQSRGLFGAIAGFI 353
58	343 RGLFGAIAGFIEGGWTG 359
59	349 IAGFIEGGWTGMIDGWY 365
60	355 GGWTGMIDGWYGYHHQN 371
61	361 IDGWYGYHHQNEQGSGY 377
62	367 YHHQNEQGSGYAADQKS 383
63	373 QGSGYAADQKSTQNAIN 389
64	379 ADQKSTQNAINGITNKV 395
65	385 ONAINGITNKVNTVIEK 401
66	391 ITNKVNTVIEKMNIQFT 407
67	397 TVIEKMNIQFTAVGKEF 413
68	403 NIQFTAVGKEFNKLEKR 419
69	409 VGKEFNKLEKRMENLNK 425
70	415 KLEKRMENLNKKVDDGF 431
71	421 ENLNKKVDDGFLDIWTY 437
72	427 VDDGELDIWTYNAELLV 443
73	433 DIWTYNAELLVLLENER 449
74	439 AELLVLLENERTLDFHD 455
75	445 LENERTLDFHDSNVKNL 461
76	451 LDFHDSNVKNLYEKVKS 467
77	457 NVKNLYEKVKSQLKNNA 473

78	463 EKVKSQLKNNAKEIGNG 479
79	469 LKNNAKEIGNGCFEFYH 485
80	475 EIGNGCEEFYHKCDNEC 491
81	481 FEFYHKCDNECMESVRN 497
82	487 CDNECMESVRNGTYDYP 503
83	493 ESVRNGTYDYPKYSEES 509
84	499 TYDYPKYSEESKLNREK 515
85	505 YSEESKLNREKVDGVKL 521
86	511 LNREKVDGVKLESMGIY 527
87	517 DGVKLESMGIYQILAIY 533
88	523 SMGIYQILAIYSTVASS 539
89	529 ILAIYSTVASSLVLLVS 545
90	535 TVASSLVLLVSLGAISF 551
91	541 VLLVSLGAISFWMCSNG 557
92	547 GAISFWMCSNGSLQCRI 563
93	553 MCSNGSLQCRICI 565

13-17-mer peptides with 11-12 amino acid overlap spanning the Hemagglutinin sequence.
